# Dietary Blueberry and Bifidobacteria Attenuate Nonalcoholic Fatty Liver Disease in Rats by Affecting SIRT1-Mediated Signaling Pathway

**DOI:** 10.1155/2014/469059

**Published:** 2014-11-27

**Authors:** Tingting Ren, Chao Huang, Mingliang Cheng

**Affiliations:** ^1^Biochemistry Department, Affiliated Hospital of Guiyang Medical College, Guiyang, Guizhou 550004, China; ^2^Guiyang Medical College, Guiyang, Guizhou 550004, China; ^3^Department of Infectious Diseases, Affiliated Hospital of Guiyang Medical College, 28 Guiyi Street, Guiyang, Guizhou 550004, China

## Abstract

NAFLD model rats were established and divided into NAFLD model (MG group), SIRT1 RNAi (SI group), blueberry juice (BJ group), blueberry juice + bifidobacteria (BJB group), blueberry juice + SIRT1 RNAi (BJSI group), and blueberry juice + bifidobacteria + SIRT1 RNAi groups (BJBSI group). A group with normal rats was a control group (CG). BJB group ameliorated NAFLD, which was better than BJ group (*P* < 0.05). The lipid accumulation was lower in CG, BJ, and BJB groups than that in MG, SI, BJSI, and BJBSI groups (*P* < 0.05). The levels of SIRT1 and PPAR-*α* were higher in CG, BJ, and BJB groups than those in MG, SI, BJSI, and BJBSI groups (*P* < 0.05). The levels of SREBP-1c were lower in CG, BJ, and BJB groups than those in MG, SI, BJSI, and BJBSI groups (*P* < 0.05). The biochemical indexes SOD, GSH, and HDL-c were improved from CG to BJB group (*P* < 0.05). Inversely, the levels of AST and ALT, TG, TC, LDL-c, and MDA were decreased from CG to BJB group (*P* < 0.05). These changes enhance antioxidative capability and biochemical index of rats. Blueberry juice and bifidobacteria improve NAFLD by activating SIRTI-mediating signaling pathway.

## 1. Introduction

Nonalcoholic fatty liver disease (NAFLD) is a progressive pathological change in chronic liver diseases and ranges from 9 to 36.9% of the population in different places of the world [1]. NAFLD is often associated with hepatocellular damage, which can be evaluated by the levels of aspartate aminotransferase (AST) and alanine aminotransferase (ALT) [2]. AST is a potential biomarker for the diagnosis of early onset of NAFLD [3]. The ratio of AST-to-platelet has also been used for the diagnosis of NAFLD patients [4]. NAFLD is closely related with risk factors such as increased concentrations of plasma triglyceride (TG) and low-density lipoprotein-cholesterol (LDL-c) [5, 6]. The ratio of TG to high-density lipoprotein-cholesterol (HDL-c) is regarded as a useful biomarker for the diagnosis of atherogenic lipid abnormalities. TG/HDL-c ratio can also be used to identify NAFLD patients with metabolic derangement [7].

Oxidative stress is one of the main causes of NAFLD, which is further exacerbated with steatohepatitis [8]. Malondialdehyde (MDA) and reduced glutathione (GSH) are biomarkers of oxidative stress [9, 10]. Under oxidative stress, intracellular superoxide species are often produced and associated with hepatic injury, and thus the injury can be prevented by the elimination of intracellular superoxide species [11]. Hepatic antioxidant enzyme, superoxide dismutase (SOD), plays a critical role in eliminating the superoxide species and is often highly expressed in hepatic injury [11]. Plasma high-density lipoprotein (HDL) is another antioxidant protein and involved in antiatherogenic effects. Plasma HDL can reduce the level of nonesterified fatty acid hydroperoxides and participates in the antioxidant property of HDL [12].

Although hepatocytes hold promising mechanisms for treating NAFLD, the medicine therapy seems necessary for the treatment of NAFLD, such as metformin, rosuvastatin, and pioglitazone [13, 14]. However, most of the medicine has unwanted adverse effects [15, 16] and limits its usage. Therefore, it is critical to find an affective therapeutic way with fewer side effects [16]. Nonpharmaceutical therapy has become a new trend for the therapy of NAFLD [17]. Blueberry (*Vaccinium ashei*), a kind of small fruits, may meet such a demand and has pronounced health effects on NAFLD due to being rich in antioxidants [18, 19]. Our previous results show that dietary supplementation of blueberry can enhance antioxidative capability of the liver via increasing MT expression and SOD activity, resulting in promoting HSC inactivation and thus decreasing matrix collagen accumulation in hepatic cells and therefore ameliorating hepatic fibrosis [20]. However, the mechanisms of blueberry juice for improving the hepatic injury are still widely unknown, and the therapeutic effects are still needed to be improved. Some work reported that bifidobacteria possess strong antioxidant activity [21, 22]. Blueberry and bifidobacteria also show higher protective effects on hepatocytes injury. They reduce the liver injury and improve the barrier functions and antioxidant activity [23].

To understand the molecular mechanisms of the action of blueberry and bifidobacteria, Sirtuins type 1 (SIRT1), an important histone deacetylase, is a potential molecule and main determinant of whole body homeostasis in mammals by regulating many transcriptional regulators in metabolic tissues such as liver and adipose tissues [24]. Sterol regulatory element binding protein (SREBP)-1c is a transcription factor for controlling the expression of genes related to fatty acid and triglyceride synthesis. SIRT1 controls the expression and function of SREBP-1c and decreases hepatic lipogenesis via inhibiting the bioactivity of SREBP-1c [25]. Furthermore, SIRT1 regulates lipid homeostasis by upregulating peroxisome proliferators-activated receptor alpha (PPAR-)*α*, a nuclear receptor mediating adaptive response such as fasting and starvation [26]. PPAR-*α* activation can enhance the protein level of antioxidant enzymes, such as SOD [27]. Blueberry and bifidobacteria may ameliorate the liver disease via SIRT1 signaling pathway and have not been explored yet. Here, we want to explore the possible mechanism for protective effects of blueberry and bifidobacteria diet on NAFLD via SIRT1-mediated pathway.

## 2. Materials and Methods

### 2.1. Materials

Blueberry juice was prepared from Guizhou Academy of Sciences (Guizhou, China). The blueberry was stored at −20°C until experimental use. Fresh blueberry juice was prepared using homogenization. Mixed cultures of bifidobacteria (*Bifidobacterium animalis, Bifidobacterium bifidum, Bifidobacterium breve, Bifidobacterium infantis,* and* Bifidobacterium longum*) were used according to a previous report [29]. All the bacteria were purchased from China General Microorganisms Culture Collection Center, Institute of Microbiology (Beijing, China).

### 2.2. High-Fat Diet- (HFD-) Induced Rat Model of NAFLD

**Figure 1:**
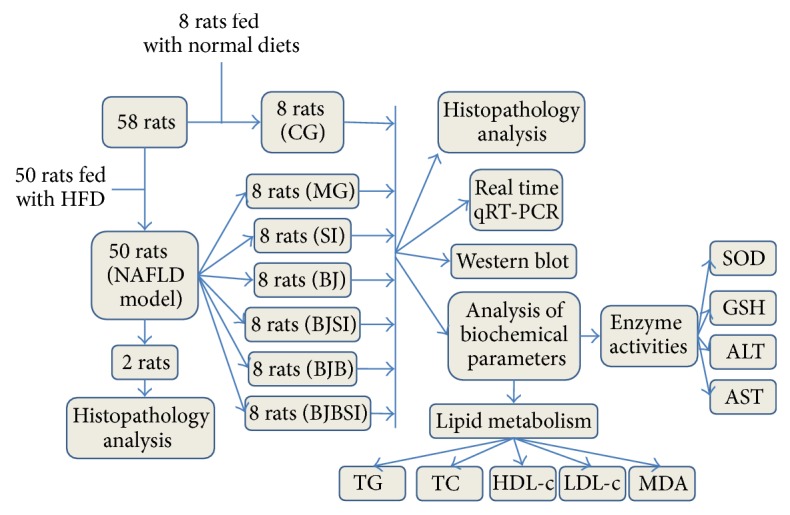
The flowchart of study. HFD: high-fat diet; NAFLD: nonalcoholic fatty liver disease; CG: a group with normal rats was assigned as a control group; MG: NAFLD model group; SI: SIRT1 RNAi group; BJ: blueberry juice group; BJSI: blueberry juice + SIRT1 RNAi group; BJB: blueberry juice + bifidobacteria group; BJBSI: blueberry juice + bifidobacteria + SIRT1 RNAi group; SOD: superoxide dismutase; GSH: reduced glutathione; AST: aspartate aminotransferase; ALT: alanine aminotransferase; TG: total triglyceride; TC: total cholesterol; HDL-c: high-density lipoprotein-cholesterol; LDL-c: low-density lipoprotein-cholesterol; MDA: malondialdehyde.

The study was designed as Figure 1 showed. Fifty-eight male Sprague-Dawley rats (200 to 250 g) were purchased from the Animal Center of Guiyang Medical College (Approval Number SCXK (Guizhou) 2002-0001, Guiyang, China). After the acclimatization, 50 rats were randomly selected and fed with a HFD for 8 weeks. The high-fat diet was prepared according to a previous report [30]. The control diet was composed of 4.3 percent fat (10 percent of the metabolizable energy), and the high-fat diet was composed of 35 percent fat (31.6 percent saturated fat and 3.2 percent unsaturated fat, 57 percent of the metabolizable energy). The established rat model was evaluated in two rats using histopathological method and the 48 remaining rats were randomly divided into six groups (8 animals/group) as Figure 1 showed. The remaining 8 normal rats were assigned as a control group (CG group) (Figure 1).

### 2.3. Experimental Design

The pshRNA-H1-Luc lentivector was purchased from System Biosciences (Mountain View, USA). The siRNA sequence targeting SIRT1, 5′-CAGGTTGCAGGAATCCAAAG-3′ was synthesized (Takala (Dalian) Co. Ltd., Dalian, China) and cloned into the pshRNA-H1-Luc lentivector, namely, pshRNA-H1-Luc-SIRT1-siRNA. The reconstructed vectors were cotransfected into 293T producer cells using Lipofectamine 2000 (Invitrogen, Shanghai, China) and pPACK Packaging Plasmid Mix (System Biosciences, Mountain View, USA). Viral supernatants were collected after 48 h transfection. The titers were measured using serial dilutions of lentivirus. Twenty-four model rats with NAFLD were intra-articularly injected with 0.1 mL culture medium containing the LV-mediated SIRT1 siRNA. As Figure 1 showed, normal rats (without NAFLD) were assigned as a control group (CG group) and NAFLD model rats were divided into model group (MG group), blueberry juice group (BJ group), SIRT1 RNAi group (SI group), blueberry juice + bifidobacteria group (BJB group), blueberry juice group + SIRT1 RNAi group (BJSI group), and blueberry juice + bifidobacteria + SIRT1 RNAi group (BJBSI group). Rats in CG, MG, and SI groups were fed with a normal diet and water. Blueberry juice (15 g/kg, once a day) or blueberry juice + bifidobacteria (each rat received 3 mL (10^8^ colony forming units [CFU]/mL) of the bifidobacteria, once a day) [23] were given to the rats in corresponding groups. After eight weeks, rats were killed for collecting blood and livers. Each liver was fixed in 10% neutral formalin. The remains of liver were stored at −80°C. Rat serum was prepared using centrifugation at 1500 rpm for 15 min and also stored at −80°C. All the procedures for animal studies were consistent with the Animal Care and Use Guidance of Guiyang Medical College (Guiyang, China).

### 2.4. Hepatic Lipid Accumulation Assay

A total 10 mg of each liver sample in 2 mL PBS was gently passed through a 16-gauge blunt-end needle (Catalog Number 28110, Beijing Biocoen Biotechnology Co., Ltd., Beijing, China) for three times. A single cell suspension was made via filtration through a 100 *μ*m cell strainer. Hepatic lipid accumulation assay was performed using Hepatic Lipid Accumulation Kit (Catalog Number ab133131), which was purchased from Abcam Trading (Shanghai) Company Ltd. (Shanghai, China). Briefly, hepatocytes were prepared at a density of 10^4^ cells/well in a 96-well plate and stained with Oil Red O and examined by measuring the absorbance at 490 nm.

### 2.5. Histopathology Analysis

Liver samples were embedded in paraffin after 24 h fixation in 10% formalin. Subsequently, samples were cut into 5 mm pieces and placed on the slides. All samples were then stained with hematoxylin and eosin (H&E) and Oil Red O to evaluate the results of NAFLD [31–33]. After deparaffinization and subsequent rehydration and antigen unmasking, liver sections were treated with 3% H_2_O_2_ for 10 min. They were then incubated with anti-SIRT1 (ab104833, Abcam, Cambridge, USA), anti-SREBP-1c (ab3259, Abcam), and anti-PPAR-*α* (ab8934, Abcam) antibody (1 : 100) overnight at 4°C. Samples were then processed using an EnVision kit (Production Number 10N1775A, Dako company, Dako, Denmark) while PBS was used as a negative control. The cells were considered to be positive when cytoplasm/nucleus was stained with brown staining. All the images were analyzed by the Biomias 2000 Image Instrument.

### 2.6. Real-Time Quantitative Reverse Transcriptase-Polymerase Chain Reaction (qRT-PCR)

Total RNA was extracted from liver tissues using RNA Extraction kit ver4.0 (TaKaRa Biotechnology (Dalian) Co., Ltd., Dalian, China). The RNA was reversely transcripted into cDNA using M-MuLV reverse transcriptase + oligo (dT) primers. The SYBR Green DNA PCR kit (Production Number 0804104, Applied Biosystems, Foster City, USA) was used for real-time RT-PCR analysis. PCR primers are as follows: SIRT1, F: 5′AGGGAACCTCTGCCTCATCTAC3′, R: 5′GGCATACTCGCCACCTAACC3′; SREBP-1c, F: 5′CGCTACCGTTCCTCTATCAATG3′, R: 5′CGTTTCTACCACTTCAGGTTTCA3′; PPAR-*α*, F: 5′ATTTGCCAAGGCTATCCCA3′, R: 5′CAGCATCCCGTCTTTGTTCA3′; and GAPDH, F: 5′GGAAAGCTGTGGCGTGAT3′, R: 5′AAGGTGGAAGAATGGGAGTT3′; the values of cycle time for the interest genes were normalized with GAPDH. The mRNA levels were showed as relative increases comparing with the control set as 100%.

### 2.7. Western Blot Analysis

Western blot was performed as previously reported [34]. Briefly, the livers were isolated from rats under aseptic conditions. The liver lysate of samples was electrophoresed into SDS-PAGE and transferred electrophoretically to a Polyvinylidene Difluoride (PVDF) (Beijing Starget Chemicals Co., Ltd., Beijing, China). The membrane was incubated with anti-SIRT1 (ab104833, Abcam, Cambridge, USA), anti-SREBP-1c (ab3259, Abcam), and anti-PPAR-*α* (ab8934, Abcam) antibody (1 : 100) overnight at 4°C. Subsequently, all the samples were incubated with peroxidase-conjugated rabbit anti-rat IgG (1 : 2000, Shanghai Sangon Biological Engineering Technology & Services Co., Ltd, Shanghai, China). The immunoreactive band was visualized with DAB (Thermo Fisher Scientific Inc., Rockford, IL, USA).

### 2.8. Analysis of Biochemical Parameters of Enzyme Activities

NAFLD is associated with increased oxidative stress in mammals [35]. The levels of SOD, AST, ALT, and GSH have been reported as the biomarkers of oxidative stress [36–41]. Therefore, the levels of all these molecules were measured in blood samples. The serum activities of AST and ALT were determined using another biochemical analysis instrument (Siemens Advia 1650, Bensheim, Germany). Liver samples were homogenated using liquid nitrogen. The activity of SOD was measured according to a previous report [42]. A fluorometric method was used to determine the levels of GSH [43].

### 2.9. Analysis of Biochemical Parameters of Lipid Metabolism

The hallmark of NAFLD is hepatic lipid accumulation, which is closely related with the metabolism of TG, TC, HDL-c, and LDL-c [44, 45]. The blood samples were used for the measurement of TG, TC, HDL-c, and LDL-c levels, according to the commercial instructions for the biochemical analyzer (Beijing Jinji Aomeng Co., Ltd., Beijing, China). Malondialdehyde (MDA), one kind of the biomarkers of oxidative damage, was measured according to a previous report [46].

### 2.10. Statistical Analysis

Data analysis was carried out using SPSS 20 package (Chicago, IL, USA). Before statistical analysis, histograms and the Kolmogorov-Smirnov methods were performed; this determined a normal distribution of the variables. Quantitative data with normal distribution were expressed as mean ± SD and subjected to one-way analysis of variance, followed by Tukey's Post Test for multiple comparisons. Ordinal data were analyzed by Radit analysis. *P* < 0.05 was considered statistically significant.

## 3. Results

### 3.1. Blueberry Juice and Bifidobacteria Consumption Attenuates NAFLD

**Figure 2:**
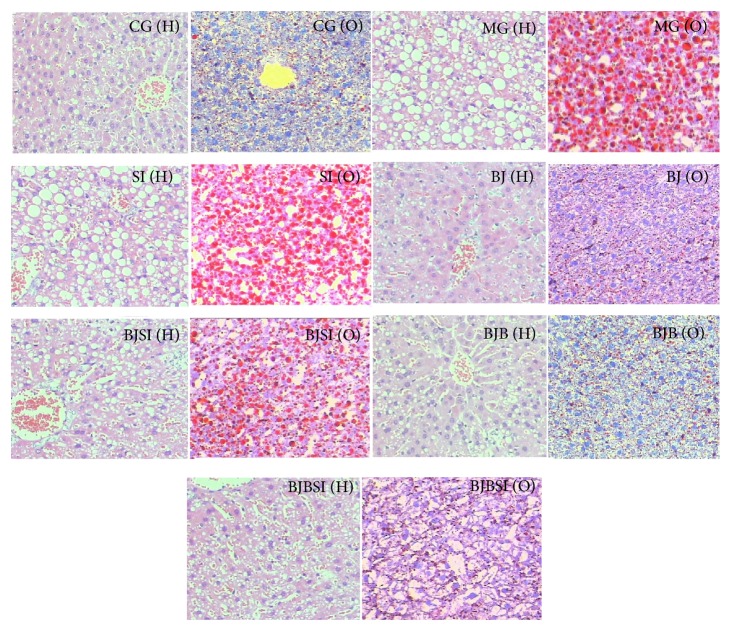
Effect of blueberry juice and bifidobacteria on NAFLD and liver damage. NAFLD rat model was induced with high-fat diet according to a previous report [28]. The nutritional contents of diets were consistent with the previous report [28]. Subsequently, some rats were feeding with or without daily blueberry juice (or blueberry and bifidobacteria) gavage. Half of the rats fed with blueberry or blueberry and bifidobacteria were transfected with SIRT1 RNAi. After 8 weeks, all samples were then stained with hematoxylin and eosin (H&E) (Marked as “H” in the figure) or Oil Red O (Marked as “O” in the figure) to evaluate the degrees of NAFLD (200x). CG (H): in control group, hepatocytes were arranged radially around a central vein and the basic structure of hepatic lobule portal area could be clearly visible. A few small-size cavities could be observed. CG (O): in control group, the number and size of red lipid droplets were very small. MG (H): in NAFLD model group, the structure of hepatic cords was heavily destroyed and many large-size cavities appeared. MG (O): in NAFLD model group, many large-size red lipid droplets were clearly visible in the adipose cells. SI (H): in SIRT1 RNAi group, the structure of hepatic cords was also heavily destroyed and many large-size cavities appeared. SI (O): in SIRT1 RNAi group, many large-size red lipid droplets were also clearly visible in the adipose cells. BJ (H): in blueberry juice group, the destroyed hepatic cords were basically repaired and orderly arranged around a central vein. A few small-size cavities could be observed. BJ (O): in blueberry juice group, many small-size red lipid droplets appeared. BJSI (H): in blueberry juice and SIRT1 RNAi group, the structure of hepatic cords was destroyed and middle-large cavities appeared. BJSI (O): in blueberry juice and SIRT1 RNAi group, many middle-large red lipid droplets appeared. BJB (H): in blueberry juice and bifidobacteria group, the destroyed hepatic cords were repaired completely and neatly arranged around a central vein. Only a few small cavities could be observed. BJB (O): in blueberry juice and bifidobacteria group, a few small-size red lipid droplets were observed. BJBSI (H): in blueberry juice and bifidobacteria and SIRT1 RNAi group, the structure of hepatic cords was destroyed. Some small- and middle-large cavities appeared. BJBSI (O): in blueberry juice and bifidobacteria and SIRT1 RNAi group, many small-size red lipid droplets were observed.

We established a rat model with NAFLD and the degree of NAFLD was assessed by H&E (marked as “H”) and Oil Red O (marked as “O”) staining (Figure 2). In CG group, hepatocytes were found with a common radial array encircling the central veins, and no large lipid droplets were observed. In NAFLD MG group, the lobular structures of hepatocytes were disrupted, and the hepatic plates were diffused with many lipid droplets. In SI group, the lobular structures of hepatocytes were significantly disrupted, and the hepatic plates were diffused with more lipid droplets. These histopathological varieties showed that a NAFLD rat model was established successfully. The degrees of NAFLD were significantly alleviated and degenerated hepatocyte fat was reduced in BJ group and BJB groups compared with those in MG, SI, BJSI, and BJBSI groups (Figure 2). In SI group, the structure of hepatic cords was destroyed and many lipid droplets appeared when the gene SIRT1 was silenced, which was similar with that in MG group. In BJ group, the hepatic cords could be observed and orderly arranged around a central vein. A few small-size lipid droplets could be observed. In BJSI group, the structure of hepatic cords was destroyed and many lipid droplets appeared when the gene SIRT1 was silenced. In BJB group, the hepatic cords were clearly observed and neatly arranged around a central vein and a few lipid droplets appeared. In BJBSI group, the structure of hepatic cords was destroyed. Some small- and middle-large lipid droplets appeared. Hepatic lipid accumulation assay showed the similar changing trend for degrees of fat accumulation from CG to BJBSI groups. The results showed that blueberry juice and bifidobacteria consumption can improve the symptoms of NAFLD significantly compared with the group only fed with blueberry juice.

### 3.2. Effects of Blueberry Juice and Bifidobacteria on Hepatic Lipid Accumulation Assay

**Figure 3:**
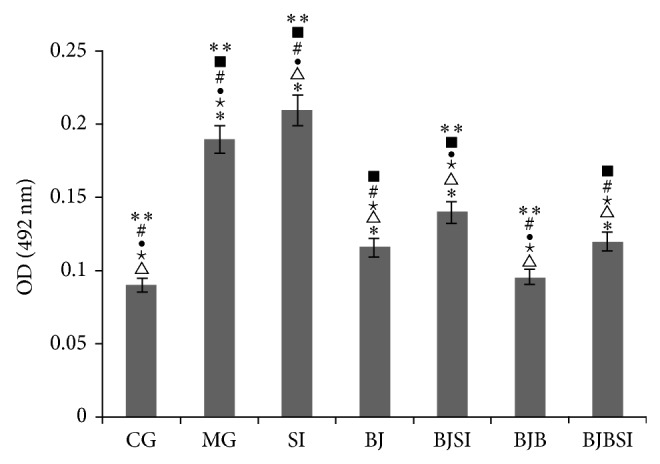
Lipid accumulation in normal rats and NAFLD model rats. CG: a group with normal rats was assigned as a control group; MG: NAFLD model group; SI: SIRT1 RNAi group; BJ: blueberry juice group; BJSI: blueberry juice + SIRT1 RNAi group; BJB: blueberry juice + bifidobacteria group; BJBSI: blueberry juice + bifidobacteria + SIRT1 RNAi group. ^*^ *P* < 0.05 versus a CG group; ^△^ *P* < 0.05 versus a MG group; ^★^ *P* < 0.05 versus a SI group; ^●^ *P* < 0.01 versus a BJ group; ^#^ *P* < 0.05 versus a BJSI group; ^■^ *P* < 0.05 versus a BJB group; ^**^ *P* < 0.05 versus a BJBSI group. All the data were presented as mean ± SD (*n* = 8).

Analysis of hepatic lipid accumulation showed that high levels of lipids were also found in MG, SI, BJSI, and BJBSI groups compared with those in CG, BJ, and BJP groups (*P* < 0.05, Figure 3). SIRT1 RNAi increased the lipid levels compared to a model group (*P* < 0.05, Figure 3). The result suggested that blueberry juice and bifidobacteria affects lipid levels. The combination of blueberry juice and bifidobacteria significantly controlled lipids levels, which were similar with those from a control group.

### 3.3. Histopathology Analysis

**Figure 4:**
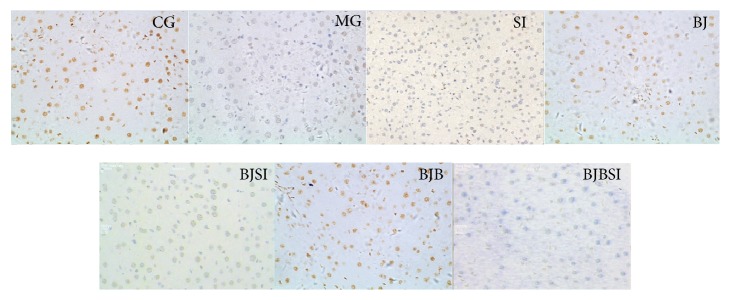
Immunohistochemistry analysis for the effect of blueberry juice and bifidobacteria on SIRT1 expression in NAFLD rat livers (200x). CG: in control group, SIRT1 was highly expressed in nucleus with brown color. MG: in NAFLD model group, SIRT1 was lowly expressed with very light brown color. SI: in NAFLD model group with SIT1 RNAi, SIRT1 was little expressed with light blue color. BJ: in blueberry juice group, SIRT1 was highly expressed in nucleus with brown color. BJSI: in blueberry juice and SIRT1 RNAi group, the expression of SIRT1 was significantly inhibited with very light brown color. BJB: in blueberry juice and bifidobacteria group, SIRT1 was highly expressed in nucleus with brown color. BJBSI: in blueberry juice and bifidobacteria and SIRT1 RNAi group, the expression of SIRT1 was completely inhibited with blue color.

**Figure 5:**
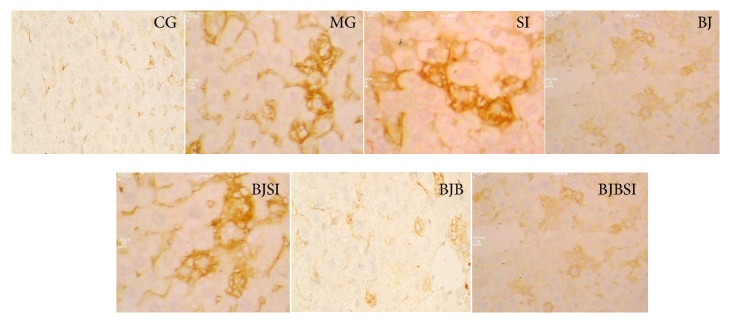
Immunohistochemistry (IHC) analysis for the effect of blueberry juice and bifidobacteria on SREBP-1c expression in NAFLD rat livers (200x). CG: in control group, SREBP-1c was lowly expressed with light brown color. MG: in NAFLD model group, SREBP-1c was highly expressed with dark brown color. SI: in NAFLD model group with SIRT1 RNAi, SREBP-1c was highly expressed with dark brown color. BJ: in blueberry juice group, SREBP-1c was lowly expressed with light brown color. BJSI: in blueberry juice and SIRT1 RNAi group, the expression of SREBP-1c was significantly enhanced with dark brown color. BJB: in blueberry juice and bifidobacteria group, SREBP-1c was lowly expressed with light brown color. BJBSI: in blueberry juice and bifidobacteria and SIRT1 RNAi group, the expression of SREBP-1c was significantly enhanced with dark brown color.

**Figure 6:**
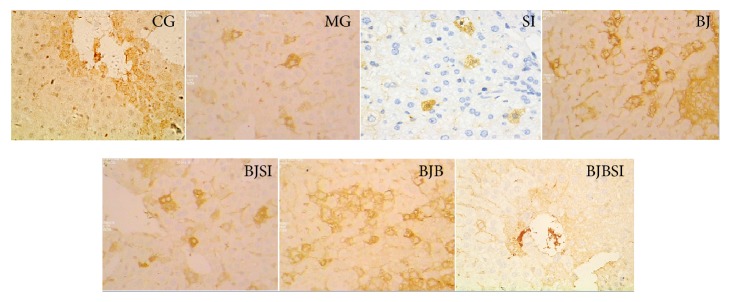
Immunohistochemistry (IHC) analysis for the effect of blueberry juice and bifidobacteria on PPAR-*α* expression in NAFLD rat livers (200x). CG: in control group, PPAR-*α* was highly expressed in cytoplasm with brown color. MG: in NAFLD model group, PPAR-*α* was lowly expressed in cytoplasm with very light brown color. SI: in NAFLD model group with SIRT1 RNAi, PPAR-*α* was lowly expressed in cytoplasm with very light brown color. BJ: in blueberry juice group, PPAR-*α* was highly expressed in cytoplasm with brown color. BJSI: in blueberry juice and SIRT1 RNAi group, the expression of PPAR-*α* was significantly inhibited with very light brown color. BJB: in blueberry juice and bifidobacteria group, PPAR-*α* was highly expressed in cytoplasm with brown color. BJBSI: in blueberry juice and bifidobacteria and SIRT1 RNAi group, the expression of PPAR-*α* was completely inhibited with blue color.

Immunohistochemistry staining indicated that SIRT1 was expressed in nucleus (Figure 4). SIRT1-positive staining was restricted to hepatic cells in control, BJ, and BJB groups, and a significantly stronger immune staining was noted in these groups. In MG, SI, BJSI, and BJBSI groups, SIRT1 was weaker immune staining with very light brown color. Compared with SIRT1, SREBP-1c was expressed in cytoplasm (Figure 5). SREBP-1c-negative staining was restricted to hepatic cells in CG, BJ, and BJB groups, and a significantly weaker immune staining was noted in these groups. In MG, SI, BJSI, and BJBSI groups, SREBP-1c was stronger immune staining with very light brown color. Just like SREBP-1c, PPAR-*α* was also expressed in cytoplasm (Figure 6). In contrast, PPAR-*α*-positive staining was restricted to hepatic cells in CG, BJ, and BJB groups, and a significantly stronger immune staining was noted in these groups (Figure 6). In MG, SI, BJSI, and BJBSI groups, PPAR-*α* was weaker immune staining with very light brown color (Figure 6). All the results suggested that SIRT1 can upregulate the expression of PPAR-*α* and downregulate the level of SREBP-1c.

### 3.4. Effects of Blueberry Juice and Bifidobacteria on the mRNA Levels of SIRT1, SREBP-1c, and PPAR-*α*

**Figure 7:**
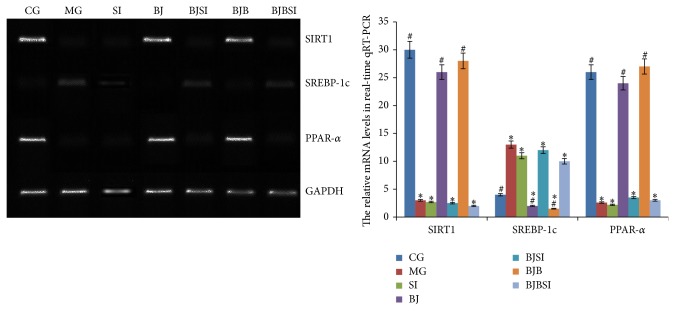
Effect of blueberry juice and bifidobacteria on mRNA levels of SIRT1, SREBP-1c, and PPAR-a in NAFLD rat livers. In control group (CG), the mRNA levels of SIRT1 and PPAR-*α* were high while the level of SREBP-1c was low. In NAFLD model group (MG) or model group with SIRT1 RNAi (SI), the mRNA levels of SIRT1 and PPAR-*α* were low while the level of SREBP-1c was high. In blueberry juice group (BJ), the mRNA levels of SIRT1 and PPAR-*α* were high while the level of SREBP-1c was low. In blueberry juice and SIRT1 RNAi group (BJSI), the mRNA levels of SIRT1 and PPAR-*α* were very low while the level of SREBP-1c was high. In blueberry juice and bifidobacteria group (BJB), the mRNA levels of SIRT1 and PPAR-*α* were very high while the level of SREBP-1c was low. In blueberry juice and bifidobacteria and SIRT1 RNAi group (BJBSI), the mRNA levels of SIRT1 and PPAR-*α* were very low while the level of SREBP-1c was high. Error bars indicate SD (*n* = 8). ^*^ *P* < 0.05 versus the control group, ^#^ *P* < 0.05 versus the model group.

We investigated the effect of blueberry on the mRNA levels of SIRT1 [47], SREBP-1c, and PPAR-*α* [48], which are biomarkers of NAFLD and activation of hepatic stellate cells (HSCs) [49]. The mRNA levels of SIRT1 and PPAR-*α* were significantly increased in CG, BJ, and BJB groups compared with those in MG, SI, BJSI, and BJBSI groups (*P* < 0.05, Figure 7). Comparatively, the levels of SREBP-1c were significantly decreased in CG, BJ, and BJB groups compared with those in MG, SI, BJSI, and BJBSI groups (*P* < 0.05, Figure 7). All the results suggested that blueberry juice and bifidobacteria affected the mRNA levels of SIRT1, SREBP-1c, and PPAR-*α*.

### 3.5. Effects of Blueberry Juice and Bifidobacteria on the Protein Levels of SIRT1, SREBP-1c, and PPAR-*α*

**Figure 8:**
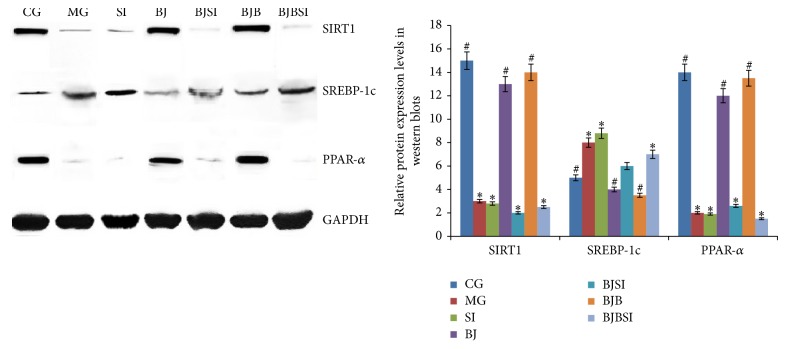
Effect of blueberry juice and bifidobacteria on protein expression of SIRT1, SREBP-1c, and PPAR-a in NAFLD rat livers. In control group (CG), the protein levels of SIRT1 and PPAR-*α* were high while the level of SREBP-1c was low. In NAFLD model group (MG) or model group with SIRT1 RNAi (SI), the protein levels of SIRT1 and PPAR-*α* were low while the level of SREBP-1c was high. In blueberry juice group (BJ), the protein levels of SIRT1 and PPAR-*α* were high while the level of SREBP-1c was low. In blueberry juice and SIRT1 RNAi group (BJSI), the protein levels of SIRT1 and PPAR-*α* were very low while the level of SREBP-1c was high. In blueberry juice and bifidobacteria group (BJB), the protein levels of SIRT1 and PPAR-*α* were high while the level of SREBP-1c was low. In blueberry juice and bifidobacteria and SIRT1 RNAi group (BJBSI), the protein levels of SIRT1 and PPAR-*α* were very low while the level of SREBP-1c was high. Error bars indicate SD (*n* = 8). ^*^ *P* < 0.05 versus the control group, ^#^ *P* < 0.05 versus the model group.

Western blot analysis showed that high levels of SIRT1 and PPAR-*α* expression were also found in CG, BJ, and BJP groups compared with those in MG, SI, BJSI, and BJBSI groups (*P* < 0.05, Figure 8). Comparatively, the protein levels of PPAR-*α* were significantly decreased in CG, BJ, and BJB groups compared with those in MG, SI, BJSI, and BJBSI groups (*P* < 0.05, Figure 8). All the results suggested that blueberry juice and bifidobacteria affects the protein levels of SIRT1, SREBP-1c, and PPAR-*α*.

### 3.6. Effects of Blueberry Juice and Bifidobacteria on Biochemical Parameters of Enzyme Activities

Next we explored whether blueberry juice affected reactive oxidative species (ROS) in liver because ROS plays an important role in NAFLD hepatocytes damage and fibrosis formation [50, 51]. We measured the haptic tissue levels of SOD, AST, ALT, and GSH, the markers of oxidative stress [36–41]. As shown in Table 1, blueberry juice and bifidobacteria consumption significantly increased SOD and GSH levels in the liver compared with those in MG, SI, BJSI, and BJBSI groups. Although blueberry juice could also increase SOD and GSH levels, blueberry juice and bifidobacteria enhanced the levels higher than only blueberry juice did (*P* < 0.05).

Serum activities of AST and ALT were also measured to determine the degree of NAFLD. Consistent with previous histological data, blueberry juice and bifidobacteria could significantly lower serum activities of AST and ALT compared with those from MG, SI, BJSI, and BJBSI groups (Table 1). All the changes could be inhibited when SIRT1 was silenced. The results suggested that the bioactivities of these enzymes are regulated by SIRT1-mediated signaling pathway.

### 3.7. Effects of Blueberry Juice and Bifidobacteria on Biochemical Parameters of Lipid Metabolism

The analysis of biochemical parameters of lipid metabolism showed that the levels of serum TC, TG, LDL-c, and MDA were reduced significantly after blueberry juice and bifidobacteria consumption compared with MG, SI, BJSI, and BJBSI groups (*P* < 0.05) (Table 2). The levels reached the lowest level when blueberry juice and bifidobacteria were compared with a BJ group (*P* < 0.05). Blueberry juice and bifidobacteria reduced the degrees of NAFLD and the result was closely related with a decrease in serum TC, TG, LDL-c, and MDA and an increase in HDL-c (Table 2, *P* < 0.05). Thus, the ratio of TG/HDL-c was significantly decreased after blueberry juice and bifidobacteria consumption. Lipid peroxidation product was decreased and antioxidant product was increased, which significantly ameliorates the hepatic damage.

## 4. Discussion

NAFLD has been reported to be the most common chronic liver disease and heavy burden of liver-related diseases in the world [52–55]. NAFLD therapies often include lifestyle modifications and pharmaceutical therapy [56–59]. However, the long-term lifestyle modification is hard to be performed. Most of medicine has adverse effects which limit its usage [60]. Thus, it is necessary to explore the novel way with fewer side effects and high therapeutic efficiency. Blueberry is a kind of fruits and widely used in the world. Bifidobacteria have been widely used for the production of yogurt [61]. Both blueberry juice and bifidobacteria have been reported to improve the symptoms of liver disease [62, 63], so blueberry juice and bifidobacteria may be an effective way for the therapy of NAFLD.

To understand the effects of blueberry and bifidobacteria on NAFLD, hepatic injury induced by high-fat diet is a well-established animal model for studying NAFLD [64, 65]. To explore the detail molecular mechanisms of effects of blueberry and bifidobacteria on NAFLD model, sirtuins may be the best candidates for the purpose. Sirtuins belong to Class III histone/protein deacetylases and there are seven kinds of sirtuins in mammals [66]. Among the sirtuins, SIRT1 is well known and widely studied. SIRT1 can regulate protein activation by deacetylating that plays a critical role in the pathophysiology of metabolic disorders, such as NAFLD. Recent work has indicated that the protein level of SIRT expression is significantly reduced in NAFLD rats fed, and the high expression of SIRT1 prevents mice from developing NAFLD [67]. Our previous work showed that blueberry juice could increase expression of Nrf2 and HO-1 in primary hepatic stellate cells [68], which is also regulated by SIRT1-mediated pathway [69]. SIRT1 can affect the mRNA level of Nrf2, Nqo1, and HO-1 in liver and have protective functions for liver jury. Probiotics and blueberry can be beneficial to protect against hepatotoxicity [23]. Bifidobacteria interventions have been reported as a potential way for the therapy of liver disease [70]. Therefore, here we considered the function of blueberry juice and bifidobacteria for improving the symptoms of NAFLD. Meanwhile, we consider the regulation of SIRT1 for other biomarkers involved with lipid metabolism, such as PPAR-*α* and SREBP-1c [71, 72]. Here, we showed that blueberry juice and bifidobacteria significantly increased SIRT1 and PPAR-*α* expression in the liver, which prevents the progression of NAFLD, suggesting a potentially important role of SIRT1 and PPAR-*α* in blueberry juice and bifidobacteria medicating protection against NAFLD. In other words, we found the possible therapeutic mechanisms of blueberry juice and bifidobacteria for improving NAFLD by activating SIRTI-mediating signaling pathway.

For further mechanism, blueberry can increase the efficient antioxidant activity [73, 74] and the antioxidant activity of bifidobacteria has been often reported [21]. Therefore, we explored the functional synergism between blueberry juice and bifidobacteria. Here, we proved that that blueberry juice and bifidobacteria consumption significantly attenuates NAFLD, which is better than the function of blueberry juice alone if considering the biochemical parameter indexes and the changes for the levels of biomarker (*P* < 0.05) (Tables 1 and 2, Figures 2 to 7). Blueberry juice and bifidobacteria can enhance the expression of SIRT1, which upregulates the protein levels of PPAR-*α* and downregulates the levels of SREBP-1c. The changes of all these molecules can increase the levels of SOD and GSH and decrease the levels of MDA, ALT, and AST, resulting in the increase of HDL-c and decrease of TG, TC, and LDL-c. All these results suggested that the blueberry juice and bifidobacteria can improve the biochemical indexes of NAFLD by activating SIRT1-mediated pathway.

Certainly, there is still some important work which is not considered here. For example, PPARg coactivator 1*α* (PGC-1*α*) plays an important role in the sirtuin signaling pathway by modulating liver fatty acid oxidation. SIRT1 deacetylation of PGC-1*α* is necessary for acting the bioactivities of mitochondrial fatty acid oxidation genes. SIRT1 can maintain the oxidation of fatty acid at low glucose concentrations. SIRT1 is a regulator of PGC-1*α* that induces the transcription of metabolic relevant genes for the oxidation of mitochondrial fatty acid [75]. Furthermore, the intake of high-fat diets can increase the level of PGC-1*α* in rats [76]. Another example is that blueberry juice is richly armed with a source of procyanidins [77], which are more effective antioxidant agents [78]. The function of procyanidins is not unique and other functions can still be explored in the future [79]. Bifidobacteria can affect the signaling pathway of many cytokines [80] and may play a more important role in the combination therapy for NAFLD. Thus, the limitation of the work is that the precise relationship is still unclear between blueberry juice and bifidobacteria and the regulation of SIRT1-mediated signaling pathway. The detail components will be analyzed in blueberry juice and bifidobacteria in the future, which is the basis for exploring the molecular mechanisms for the functions of blueberry juice and bifidobacteria.

## 5. Conclusions

We demonstrated that the protective effect of blueberry juice and bifidobacteria for NAFLD is associated with elevated hepatic expression of SIRT1, which upregulates the protein expression of PPAR-*α* and downregulates the level of SREBP-1c. The changes can increase the activity of SOD and GSH and decrease the activity of ALT and AST, which results in the increase of HDL-c and the decrease of TG, TC, LDL-c, and MDA. All these changes reduce oxidative stress in NAFLD rat and improve the symptoms. We therefore propose that dietary supplementation of blueberry juice and bifidobacteria can augment antioxidative capability of the liver presumably via stimulating SIRT1-mediated signaling pathway.

## Figures and Tables

**Table 1:** Biochemical parameters of enzyme activities for NAFLD.

Group (*n* = 10)	SOD (U/mL)	GSH (ng/L)	ALT (U/mL)	AST (U/mL)
CG	28.14 ± 3.09^△★●#**^	24.258 ± 2.147^△★#**^	46.22 ± 10.33^△★●#■^	103.55 ± 25.07^△★●**^
MG	13.25 ± 4.36^*★●#■**^	13.223 ± 1.993^*★●■**^	88.79 ± 9.48^*★●#■**^	208.24 ± 19.68^*★●#■^
SI	11.23 ± 4.15^*△●#■**^	11.178 ± 2.101^*△●#■**^	98.14 ± 10.51^*△●#■^	234.54 ± 21.56^*△●#■**^
BJ	23.34 ± 3.58^*△★#■^	22.328 ± 1.092^△★*#**^	60.45 ± 12.588^△★*#^	144.49 ± 25.288^△★#**^
BJSI	17.98 ± 2.23^*△★●■^	14.228 ± 1.631^*●■^	104.25 ± 14.48^*△★●■^	251.24 ± 17.87^*△★●■^
BJB	28.27 ± 4.79^△★●**^	25.674 ± 2.268^△★●#**^	57.94 ± 12.06^△★*#^	127.63 ± 21.32^△★#**^
BJBSI	20.25 ± 4.47^*△★■^	17.331 ± 1.694^*△★●#■^	96.58 ± 7.48^*△●■^	189.62 ± 21.36^★*●#■^

CG: a group with normal rats was assigned as a control group; MG: NAFLD model group; SI: SIRT1 RNAi group; BJ: blueberry juice group; BJSI: blueberry juice + SIRT1 RNAi group; BJB: blueberry juice + bifidobacteria group; BJBSI: blueberry juice + bifidobacteria + SIRT1 RNAi group. ^*^ *P* < 0.05 versus a CG group; ^△^ *P* < 0.05 versus a MG group; ^★^ *P* < 0.05 versus a SI group; ^●^ *P* < 0.01 versus a BJ group;^ #^ *P* < 0.05 versus a BJSI group; ^■^ *P* < 0.05 versus a BJB group; ^**^ *P* < 0.05 versus a BJBSI group. All the data were presented as mean ± SD (*n* = 8).

**Table 2:** Biochemical parameters of lipid metabolism for NAFLD.

Group (*n* = 10)	TG (nmol/L)	TC (nmol/L)	HDL (nmol/L)	LDL (nmol/L)	MDA (mmol/L)
CG	0.83 ± 0.17^△★●#**^	1.06 ± 0.48^△★#**^	1.35 ± 0.29^△★●#**^	1.03 ± 0.48^△★●#■**^	0.467 ± 0.146^△★●#**^
MG	1.79 ± 0.42^*●#■**^	2.59 ± 1.02^*★●■**^	0.67 ± 0.49^*★●■**^	1.85 ± 0.66^*^^★●■**^	1.435 ± 0.412^*★●#■**^
SI	1.85 ± 0.48^*●#■**^	2.73 ± 1.24^*△●#■**^	0.51 ± 0.32^*△●#■**^	2.15 ± 0.78^*△●#■**^	1.612 ± 0.431^*△●#■**^
BJ	1.16 ± 0.148^*△★#■**^	1.11 ± 0.89^△★#**^	1.16 ± 0.288^*△★#**^	1.55 ± 0.678^*△★#■^	0.546 ± 0.348^*△★#■**^
BJSI	2.03 ± 0.63^*△★●■**^	2.39 ± 0.63^*△★●■**^	0.77 ± 0.25^*★●■**^	1.79 ± 0.25^*★●■**^	1.132 ± 0.359^*△★●■**^
BJB	0.93 ± 0.33^△★●#**^	0.97 ± 0.54^△★●#**^	1.23 ± 0.30^△★●#**^	1.33 ± 0.58^*△★●#**^	0.441 ± 0.289^△★●#**^
BJBSI	1.36 ± 0.17^*△★●#■^	1.84 ± 0.70^*△★●#■^	0.97 ± 0.27^*△★●#■^	1.66 ± 0.47^*△★●■^	0.792 ± 0.371^*△★●#■^

CG: a group with normal rats was assigned as a control group; MG: NAFLD model group; SI: SIRT1 RNAi group; BJ: blueberry juice group; BJSI: blueberry juice + SIRT1 RNAi group; BJB: blueberry juice + bifidobacteria group; BJBSI: blueberry juice + bifidobacteria + SIRT1 RNAi group. ^*^ *P* < 0.05 versus a CG group; ^△^ *P* < 0.05 versus a MG group; ^★^ *P* < 0.05 versus a SI group; ^●^ *P* < 0.01 versus a BJ group;^ #^ *P* < 0.05 versus a BJSI group; ^■^ *P* < 0.05 versus a BJB group; ^**^ *P* < 0.05 versus a BJBSI group. All the data were presented as mean ± SD (*n* = 8).
